# Physicochemical Characterization of the Salmon Bone Hydroxyapatite and Collagen Mixture

**DOI:** 10.3390/ma19173725

**Published:** 2026-09-01

**Authors:** Francisco Muñoz, Antonia Aste, Nicole Ortega, Rosalba Escamilla, Andreu Puigdollers

**Affiliations:** 1 Laboratorio de Investigación y Desarrollo Salmoss Biotech, Santiago 7550000, Chile; antonia.aste@ug.uchile.cl (A.A.); nicole.ortega@miuandes.cl (N.O.); 2 Área de Ortodoncia, Facultad de Odontología, Universitat Internacional de Catalunya, 08017 Barcelona, Spain; andreup@uic.es; 3 Laboratorio de Oncología, Pontificia Universidad Católica de Chile, Santiago 7550000, Chile; rescamilla@uc.cl

**Keywords:** hydroxyapatite, type I collagen, salmon bone

## Abstract

Objective: To develop and characterize a particulate biomaterial composed of hydroxyapatite derived from salmon bone and bovine type I collagen (HAPS/COL), and to evaluate whether different inorganic–organic ratios influence its physicochemical properties. Materials & Methods: Biomaterials were produced using three HAPS/COL proportions (70/30, 80/20, and 90/10). Physicochemical characterization included pycnometry, X-ray diffraction (XRD), thermogravimetric analysis (TGA), and scanning electron microscopy with energy-dispersive X-ray spectroscopy (SEM-EDX). Results: Pycnometry showed densities close to that of pure hydroxyapatite, with only slight decreases attributable to collagen content. SEM-EDX images showed comparable surface morphologies characterized by hydroxyapatite particulates surrounded by collagen fibers. Ca/P ratios were slightly higher than the stoichiometric value of hydroxyapatite. XRD confirmed hydroxyapatite as the dominant crystalline phase across all formulations, with no evidence of phase alteration. TGA revealed mass losses of 2.75%, 1.90%, and 1.60% for the 70/30, 80/20, and 90/10 mixtures, respectively, with consistent mass loss-to-collagen ratios of approximately 0.09 for the 70/30 and 80/20 formulations, confirming proportional organic phase degradation. Conclusions: The three HAPS/COL ratios produced biomaterials with highly similar physicochemical profiles, indicating that within the tested range, the proportion of hydroxyapatite and collagen does not substantially alter material structure, composition, or thermal behavior. These findings confirm the successful manufacture of a stable HAPS/COL blend, allowing the use of efficient quantities of collagen, thus minimizing the costs associated with its purchase.

## 1. Introduction

Hydroxyapatite is widely used in biomedical applications due to its osteoconductive, non-toxic, and bioactive properties [1]. Its close chemical and structural similarity to human bone makes it highly suitable for promoting osteogenesis and bone regeneration. In recent years, hydroxyapatite obtained from natural sources—such as salmon bone—has gained significant attention because of its favorable biochemical characteristics, biocompatibility, and low production cost, positioning it as a promising precursor for xenograft fabrication [2]. Nonetheless, hydroxyapatite alone presents intrinsic limitations, including brittleness, low mechanical strength, and susceptibility to fragmentation, which can lead to particle migration or structural instability during surgical handling.

To overcome these disadvantages, research has increasingly focused on the development of composite biomaterials that combine inorganic minerals with organic macromolecules. This strategy follows a biomimetic rationale, as native bone is composed of approximately 70% inorganic mineral (mainly hydroxyapatite) and 30% organic matrix (predominantly Type I collagen) [3]. Reproducing this natural architecture enhances the mechanical, biological, and physicochemical performance of engineered bone substitutes. In this context, considerable progress has been made in organic–inorganic scaffolds produced through sustainable and low-energy approaches, frequently using collagen, chitosan, carrageenan, alginate, and other natural polymers as structural matrices for calcium-based fillers [4,5,6].

Type I collagen—the main protein of skin, bone, and connective tissues—is widely used in biomedical scaffolds due to its hemostatic, osteoconductive, and osteointegrative properties, its low toxicity, and its ability to support mineral deposition and cell adhesion [7,8]. Its high hydrophilicity also favors an environment that promotes cellular proliferation [9]. For these reasons, hydroxyapatite–collagen composites have been extensively investigated, typically using processes involving hydrogel formation, lyophilization, and chemical or physical cross-linking to stabilize the organic phase [10]. Despite these advances, the fabrication and characterization of biomaterials combining collagen with salmon bone hydroxyapatite (HAPS) remain insufficiently studied, and the influence of the relative proportions of collagen and mineral phase on the physicochemical characteristics of these mixtures has not been systematically evaluated. Previous studies often focus on three-dimensional scaffolds, whereas particulate formulations—potentially advantageous due to their simplicity, hydration stability, and clinical manageability—have received far less attention.

An additional and relevant unexplored aspect is the efficient use of collagen, which represents the most expensive component in hydroxyapatite and collagen formulations. Since native human bone consists of approximately a 70/30 inorganic–organic ratio and still preserve key physicochemical properties, maintaining the essential characteristics of the blend, offering a potentially cost-effective alternative without sacrificing performance [11]. The 70/30 ratio was selected to mimic the mineral-to-collagen proportion found in human bone, thereby providing a biomimetic reference. The 80/20 and 90/10 ratios were chosen to systematically reduce the organic fraction and test whether collagen content could be minimized—while maintaining the mineral as the predominant phase—without compromising the material’s physicochemical stability, structural integrity, or handling properties. We hypothesized that producing xenografts mimicking this natural proportion could require as low as 10% collagen content, like it has been studied in collagen-hydroxyapatite scaffolds, and still preserve key physicochemical properties, maintaining the essential characteristics of the blend, offering a potentially cost-effective alternative without sacrificing performance.

Therefore, collagen ratios slightly below this natural benchmark were selected to test whether reducing collagen—while maintaining the mineral majority—could still yield stable, homogeneous, and clinically workable biomaterials, measuring the effect of collagen concentration in mixture stability, water retention, particle cohesion, or structural integrity, while also assessing the potential cost reduction associated with collagen minimization.

These three ratios therefore allow us to evaluate a continuum from a biomimetic composition to formulations with substantially reduced collagen content, providing insight into the minimum organic fraction required for material functionality.

Salmon bones represent an underexploited resource for high-value biomaterial production. Fish bones obtained from the Chilean salmon industry are composed of approximately 60% hydroxyapatite, making them a valuable feedstock for calcium phosphate materials [12]. In Chile, the salmon industry ranks second globally, generating 6.371 billion U.S. dollars in 2024, with production reaching 774,531 tons in 2023, and generating significant volumes of waste [13,14].

Consequently, valorizing salmon bone waste for hydroxyapatite production presents a dual opportunity: addressing environmental challenges while creating a high-value product for bone tissue engineering applications.

Thus, the objective of the present study is to evaluate how different HAPS-collagen ratios influence the physicochemical behavior, handling properties, and structural characteristics of particulate biomaterials produced from salmon-bone-derived hydroxyapatite. This research is particularly relevant in Chile, where the salmon industry generates large quantities of bone waste, offering an underutilized and sustainable resource for xenograft production. The findings aim not only to clarify composition–property relationships in hydroxyapatite and collagen systems but also to determine whether collagen can be used more efficiently—without compromising material performance—thereby contributing to the development of more accessible, biomimetic, and economically viable bone substitutes.

## 2. Materials and Methods

### 2.1. Production of Salmon Bone Hydroxyapatite (HAPS)

The HAPS production protocol was established and optimized, using as a basis that of Muñoz et al. [2] all adhering flesh was discarded manually and with scissors. The bones were then cut and boiled for 1 h to remove any remaining matter. The bones were then kept in 0.25 M NaOH for 2 h, with stirring, to remove protein material at 20 °C. The bones were then stirred in acetone for 6 h at 25 °C to remove adhering lipid material. They were then dried in an oven for 18 h at 60 °C. The dried bones were ground in a mill. Finally, the ground material was calcined for 2 h in a muffle furnace at 1000 °C. The material was sieved to obtain a particle size between 200 and 700 microns [2].

### 2.2. Production of Collagen-Incorporated HAPS (HAPS/COL)

While collagen is commonly used in the literature to provide specific shapes to biomaterials, in 3D, for medical applications, the main objective of the present project was focused on the production of HAPS mixed with collagen (HAPS/COL) in particulate form and its physical characterization. Therefore, the results presented correspond to this specific biomaterial format. The production of HAPS/COL was based on mixing particulate HAPS in different ratios (70/30, 80/20, and 90/10) with Type I bovine collagen (COL), derived from the Achilles tendon (Merck, MT, USA), in powder form. Finally, the biomaterials were subjected to ultraviolet (UV) irradiation to induce collagen cross-linking. The samples were placed inside a UV chamber equipped with six 8-W lamps and configured to operate at a wavelength of 254 nm, which is commonly used to promote collagen photo-crosslinking. The irradiance was set to deliver a total energy dose of 10 J/cm^2^, within the operational range of the equipment (0–10 J/cm^2^). Samples were positioned at a fixed distance of 10 cm from the UV light source on the central platform of the chamber to ensure uniform exposure. Irradiation was carried out for 1 h.

### 2.3. Characterization of HAPS/COL

#### 2.3.1. X-Ray Diffraction (XRD)

The crystallinity of the samples was determined by X-ray diffraction (XRD). The analysis was carried out using a powder X-ray diffractometer (Bruker, Germany), model D8 Advance, operating at 40 kV and 30 mA with Cu Kα1 radiation. The measurement range was from 2° to 80° (2θ), with a step size of 0.02° and a counting time of 0.3 s per step. The instrument geometry corresponded to Bragg–Brentano, and a nickel filter was used. Phase identification was performed using the DIFFRAC.EVA software V 2.0 (Bruker, Germany), with the PDF-2 database.

#### 2.3.2. Thermogravimetric Analysis (TGA)

Thermogravimetric analysis (TGA) was performed to assess the thermal stability of the HAPS/COL samples. The analyses were conducted using a TGA Q50 V20.10 Build 36 system (Thermal Analysis, USA). Approximately 9 mg of each sample was heated from 50 °C to 600 °C at a heating rate of 10 °C/min. The nitrogen gas flow for the sample was set to 60 mL/min, while the balance gas flow was 40 mL/min.

#### 2.3.3. Gas Pycnometry

Gas pycnometry was used to measure the density and specific volume of the HAPS/COL samples. The analysis was performed using an AccuPyc II 1340 Series pycnometer (Micromeritics, Norcross, GA, USA). Nitrogen gas was used, with a purge fill pressure of 19.5 psig. The equilibrium rate was set to 0.005 psig/min, and five cycles were conducted for each measurement. All measurements were carried out at room temperature and performed in triplicate. The samples were analyzed as received, without any additional processing or pre-treatment.

#### 2.3.4. Scanning Electron Microscopy Coupled with Energy-Dispersive X-Ray Spectroscopy (SEM-EDX)

To evaluate the morphology of the samples along with their surface elemental composition, a Phenom Pro-X scanning electron microscope (Thermo Fisher Scientific, Waltham, MA, USA) was used. The system is equipped with a CeB_6_ filament, secondary (SE) and backscattered (BSD) electron detectors, and includes an energy-dispersive X-ray spectroscopy (EDS) analyzer. For each measurement, a small amount of sample was deposited on aluminum foil and mounted onto a 1.2 cm diameter sample holder covered with adhesive carbon film. Compressed air was used to remove any loosely attached material before inserting the sample into the instrument. Prior to imaging, the samples were sputter-coated with a thin carbon layer to improve surface conductivity and prevent charging during SEM/EDS analysis.

## 3. Results

### 3.1. XRD

Below are the spectra obtained via X-ray diffraction for the measured samples, which correspond to HAPS/COL 70/30, HAPS/COL 80/20, and HAPS/COL 90/10, reflecting the proportions used for each phase. Results for COL are also presented.

**Figure 1:**
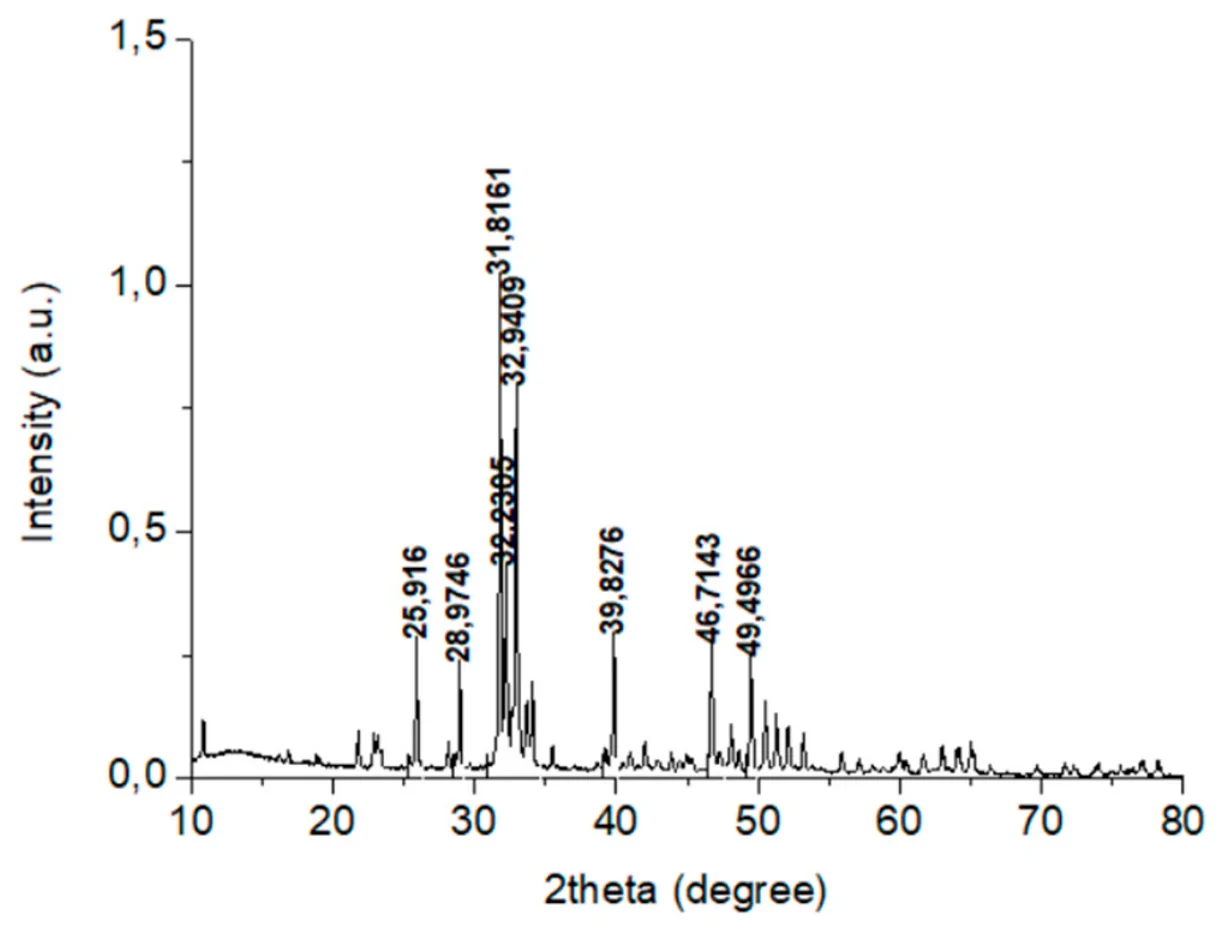
XRD pattern of HAPS/COL 70/30.

**Figure 2:**
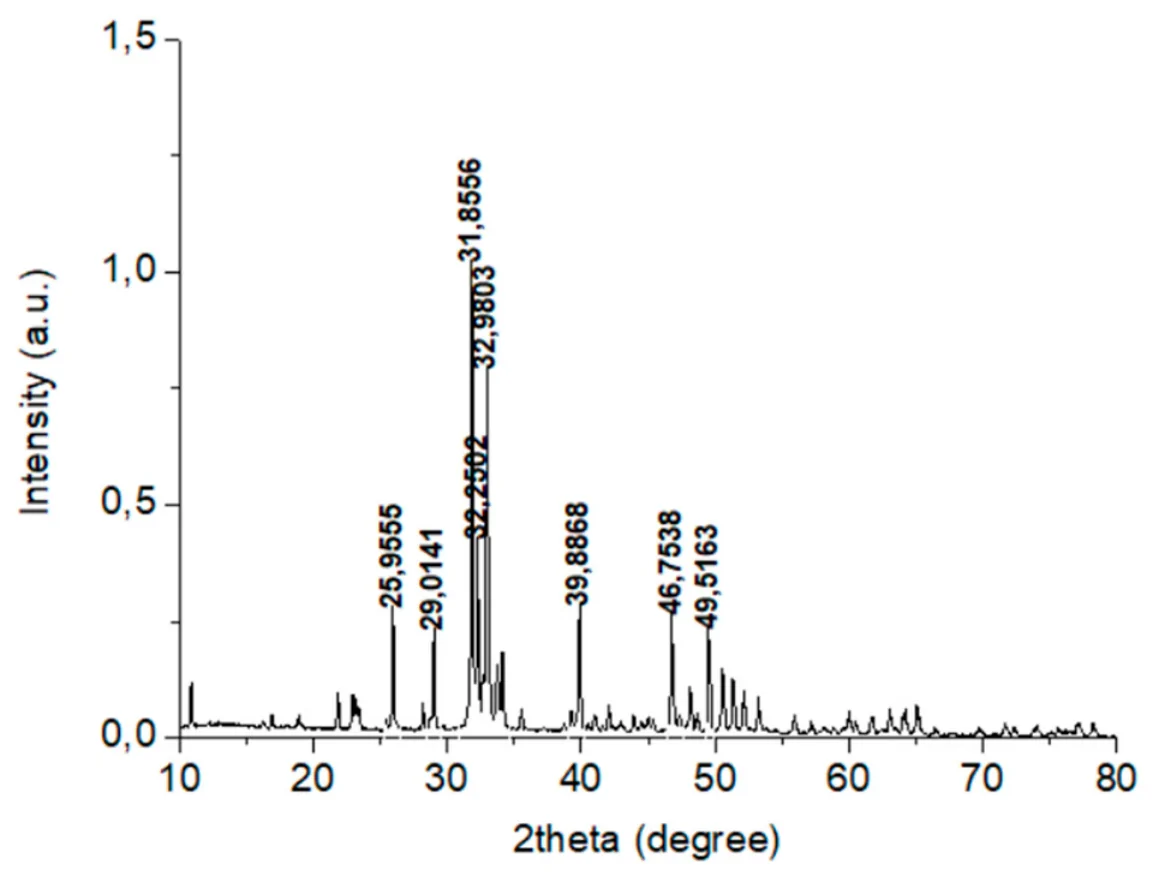
XRD pattern of HAPS/COL 80/20.

**Figure 3:**
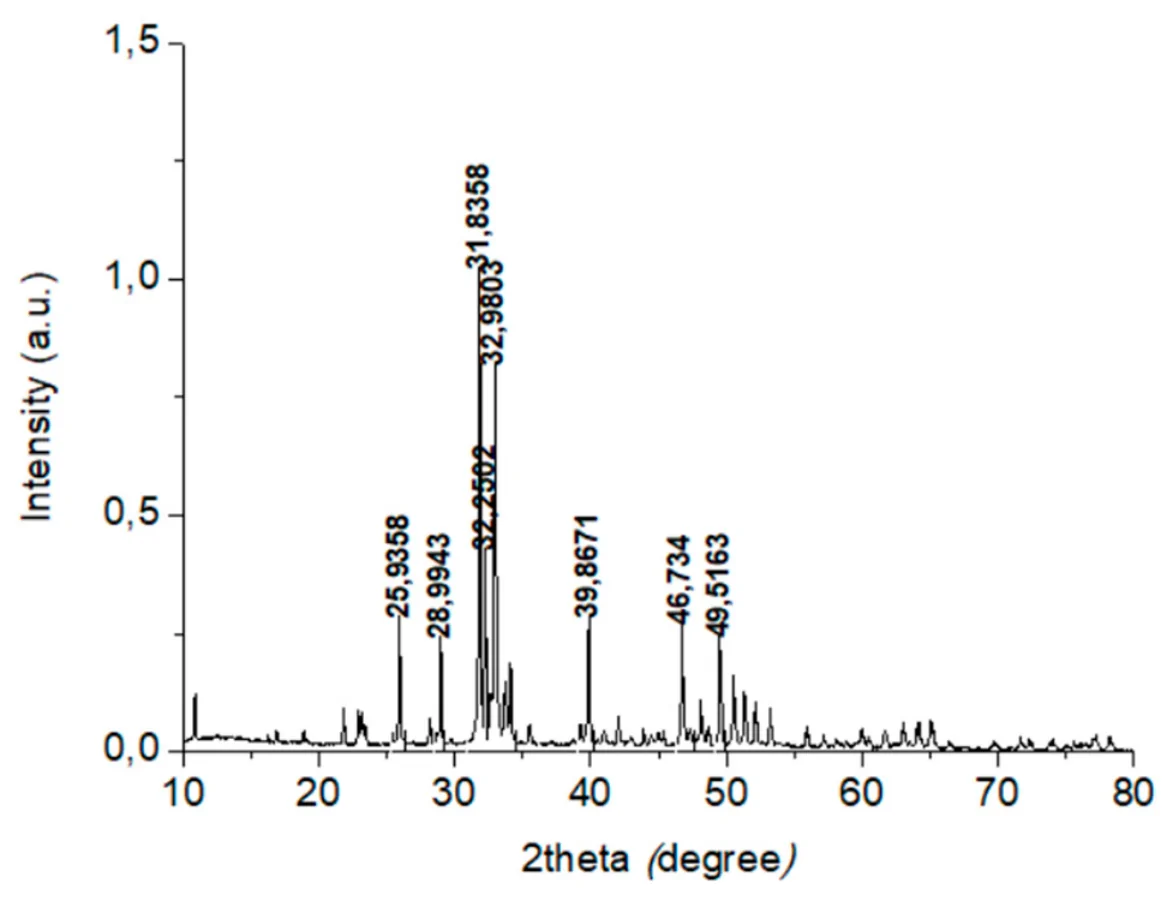
XRD pattern of HAPS/COL 90/10.

The X-ray diffraction patterns in Figure 1, Figure 2 and Figure 3 correspond to those of HAPS. Peaks are observed around the angles 25.882°, 31.765°, 32.194°, 34.062°, 39.790°, 46.693°, and 49.489°, which are characteristic of HAPS, according to the standard crystallographic plane (hkl) for this mineral [15]. The HAPS and COL mixtures were prepared in different proportions, with the inorganic phase being present in the highest quantity in all three combinations.

**Figure 4:**
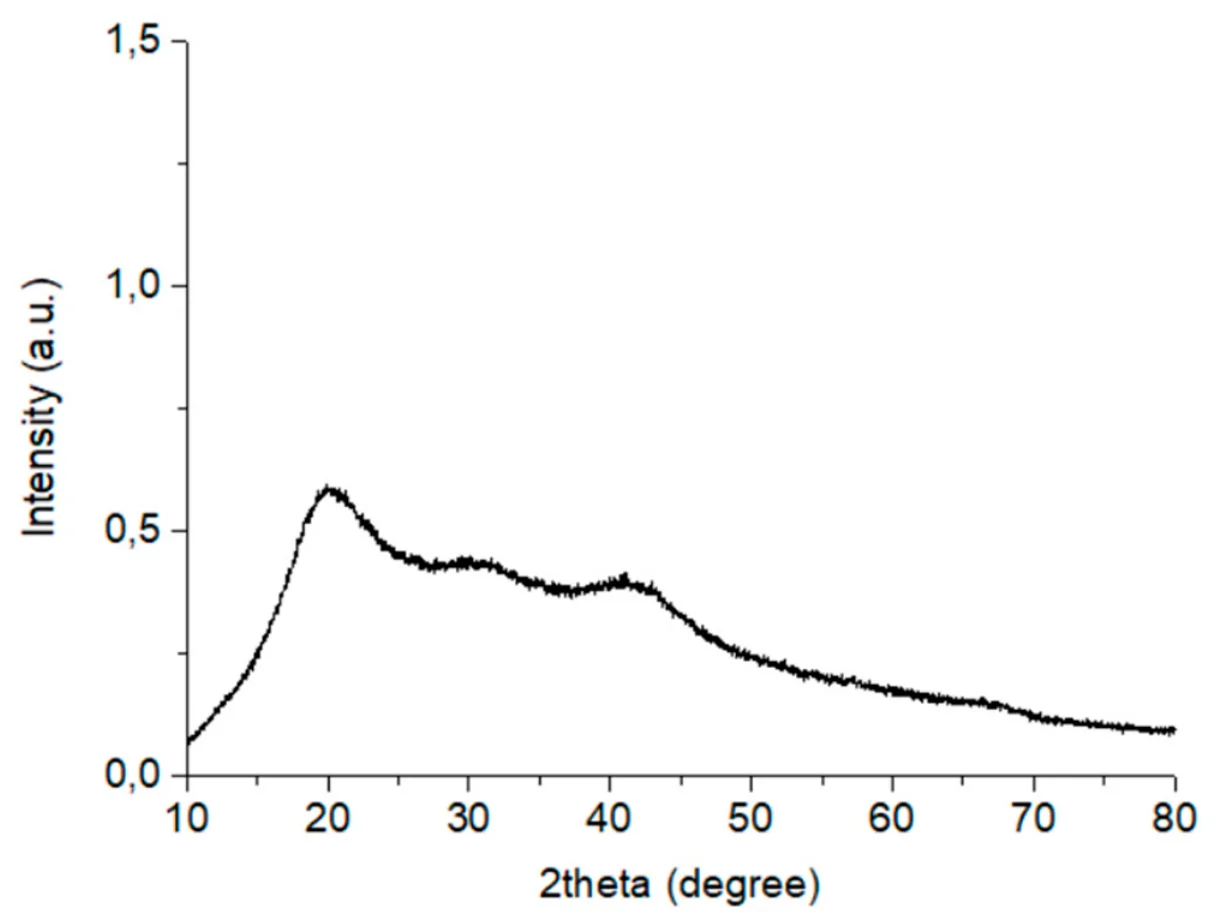
XRD pattern of COL.

Through X-ray diffraction analysis, it is possible to obtain the diffractograms of biomaterials with crystalline structures, so the results likely indicate the spectrum of the inorganic or mineral phase corresponding to HAPS, previously characterized [14]. For COL, whose diffractogram is presented in Figure 4, the result corresponds to an amorphous substance, and thus, it was not possible to identify crystalline phases. This is because the COL added to the HAPS structure was not previously crystallized, as this step is unnecessary; the goal of the present biomaterial production is to preserve as much as possible the fibrous structure of this protein.

When comparing the diffractograms of the three formulations, no noticeable differences were observed among them. In all cases, the characteristic HAPS spectrum predominated consistently, regardless of the amount of collagen incorporated into the formulation. This indicates that the variations in COL content did not modify the crystalline behavior of the mineral phase. Finally, the diffractograms obtained are consistent with those specifically obtained for hydroxyapatite and collagen composites by other authors [4,16,17].

### 3.2. TGA

**Figure 5:**
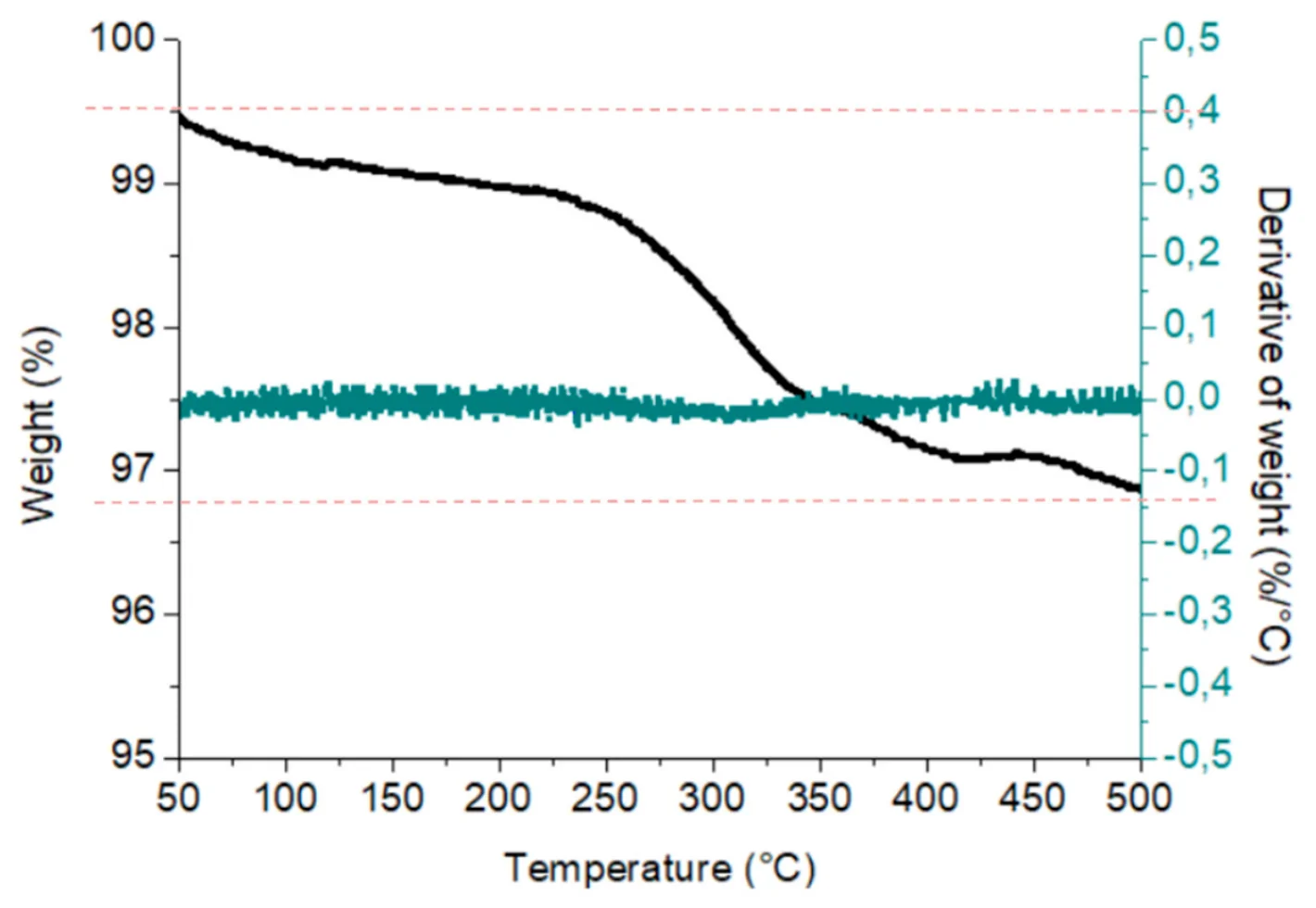
TGA of HAPS/COL 70/30.

**Figure 6:**
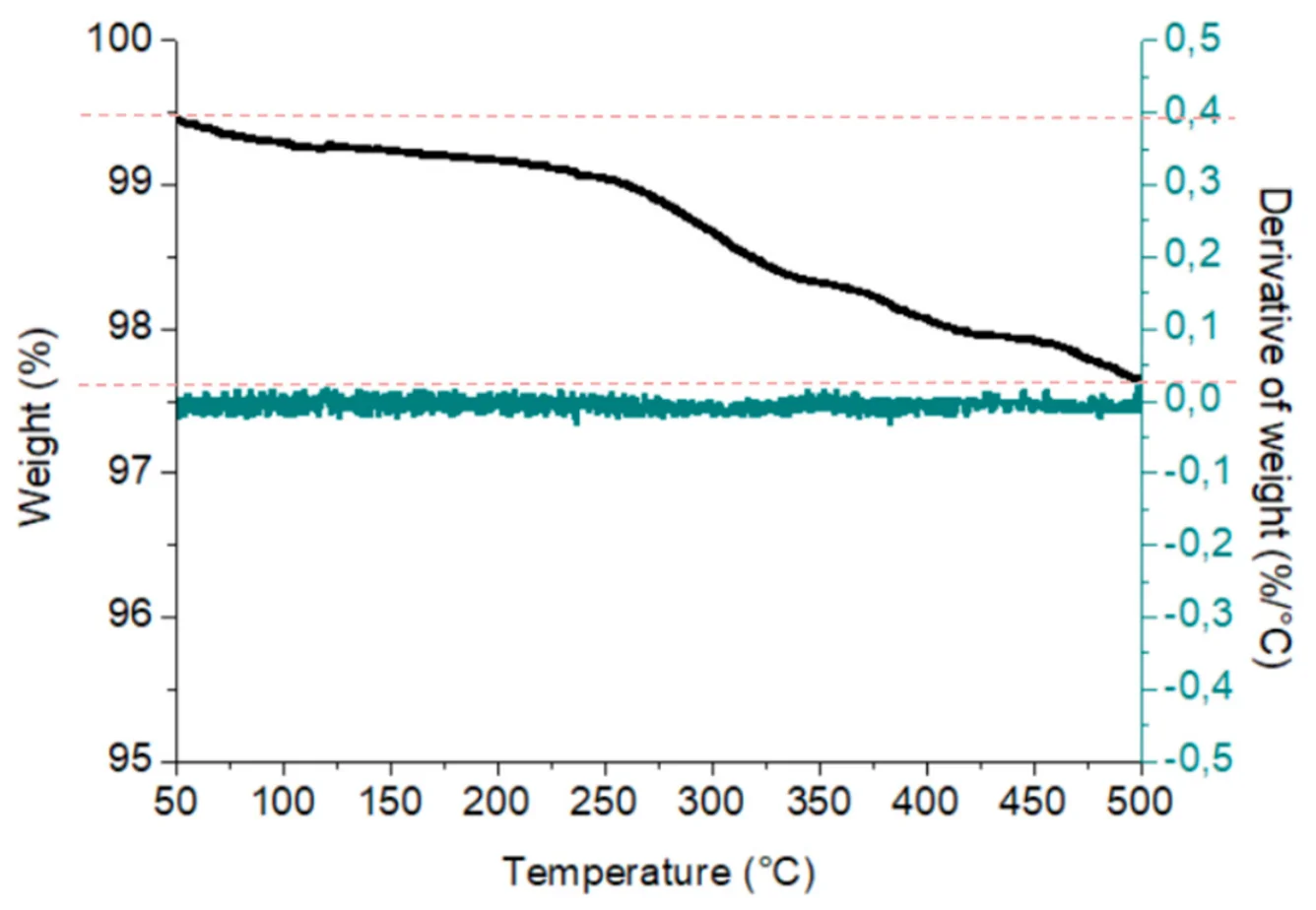
TGA of HAPS/COL 80/20.

**Figure 7:**
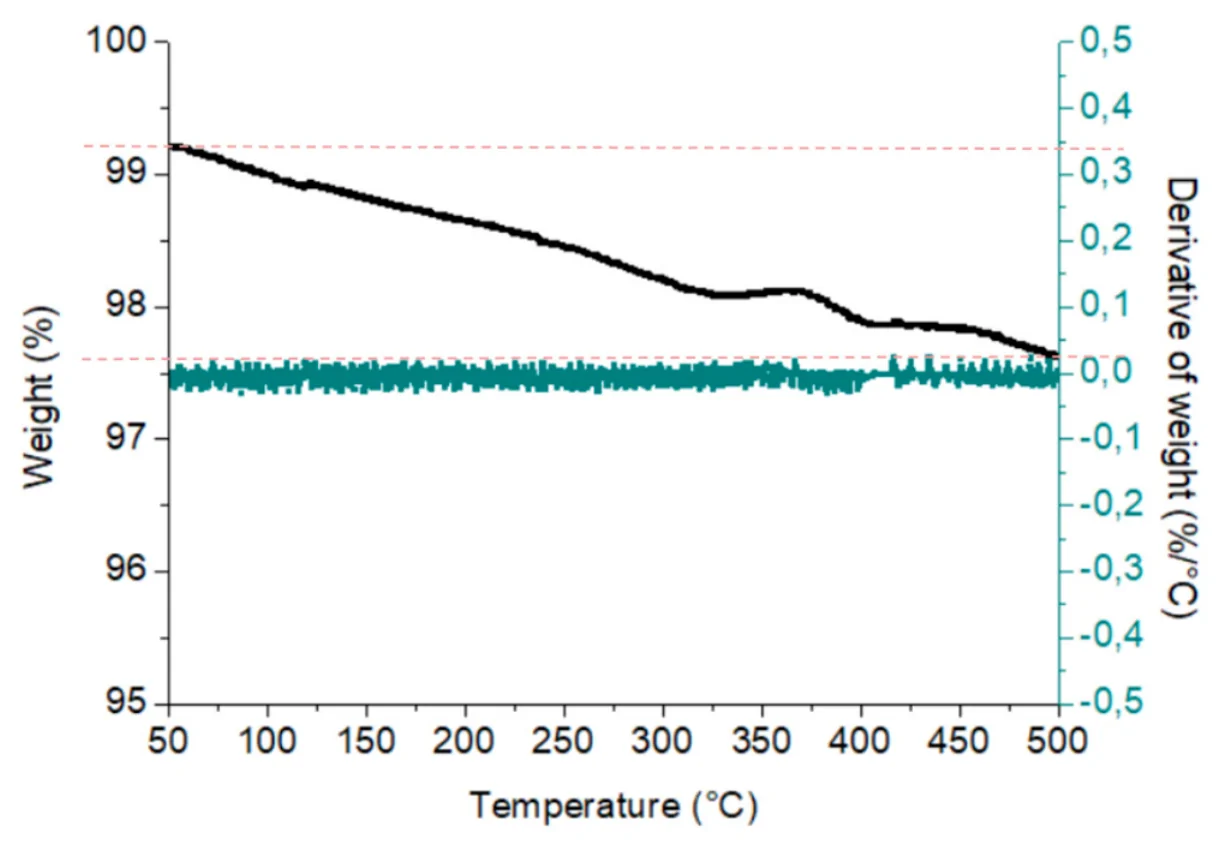
TGA of HAPS/COL 90/10.

**Figure 8:**
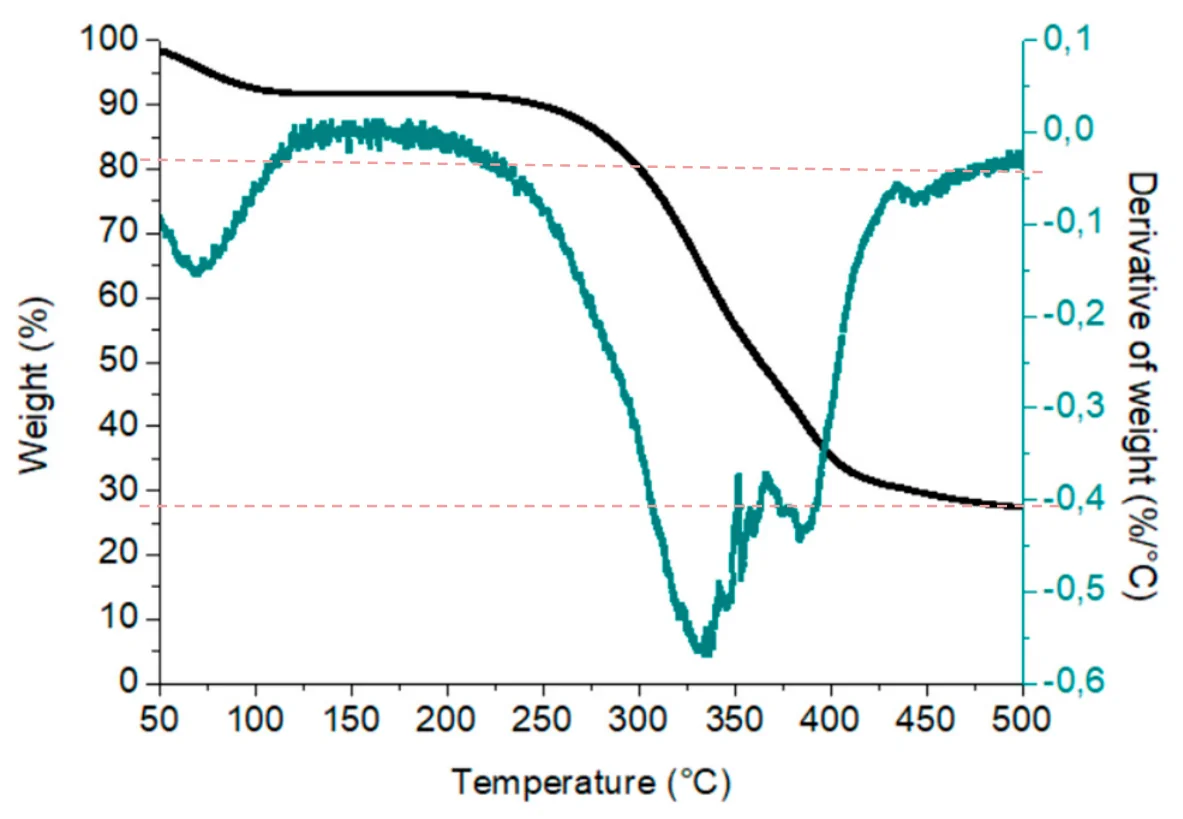
TGA of COL.

Figure 5, Figure 6 and Figure 7 present the results of the TGA performed on the biomaterials HAPS/COL 70/30, HAPS/COL 80/20, and HAPS/COL 90/10, along with the graph corresponding to COL in Figure 8.

Figure 8 shows the TGA curve of COL. A pronounced weight loss is observed between approximately 200 and 300 °C, corresponding to nearly 80% of the total sample mass. This result is consistent with findings by Durga et al. (2022) [5], who reported that weight loss in human bone—composed of hydroxyapatite and collagen—occurs in two main temperature ranges: approximately 300–500 °C and 220–600 °C, associated with the degradation of collagen and the total organic fraction, respectively.

Figure 5, Figure 6 and Figure 7 show a slight mass loss for the analyzed biomaterials. However, the sample containing the highest COL content (HAPS/COL 70/30) displays a more pronounced decline in the TGA curve. This is attributed to the larger proportion of the organic phase-COL—which exhibits lower thermal stability across the tested temperature range. Like the COL TGA curve (Figure 8), the onset and progression of weight loss typically occur between 300 and 500 °C. It is worth noting that the TGA curves for pure HAPS show no significant mass loss within this temperature range, as HAPS is free of organic matter and remains thermally stable at elevated temperatures. In contrast, the HAPS/COL 90/10 sample exhibits a more linear and less marked weight loss, consistent with its lower COL content.

From these Figures, we can identify the initial and final weight percentage of the biomaterial (indicated by the punctuated horizontal line), being 96.75%, 97.6% and 97.6% for the 70/30, 80/20 and 90/10 formulations, respectively. This way the mass loss percentage, in the analyzed 50–500 °C temperature range, for each case would be 2.75%, 1.90%, and 1.60% (Figure 5, Figure 6 and Figure 7). To compare these results, the mass loss/collagen ratio is also calculated, it being 0.092, 0.096 and 0.160 for the 70/30, 80/20 and 90/10 formulations, respectively. 

For the collagen sample, Figure 8, the 72.5% weight loss percentage is calculated from the TGA curve, where the observed residual mass is 27.5%, while DTG curve provides information for the maximum degradation temperature which is 332.4 °C.

### 3.3. Pycnometry of Gases

The results obtained by gas pycnometry are presented in Table 1.

The density values obtained were not presented linearly; however, they correspond to lower figures than those obtained for hydroxyapatite alone in previous studies. This is because the density of collagen is around 1.32 g/cm^3^ [6], which is lower than that obtained for HAPS, which corresponds to 3.16 g/cm^3^ [18]. In mixtures of hydroxyapatite and collagen, the compound biomaterial becomes less dense due to the density of the latter.

However, the non-linear relationship between collagen content and measured density suggests that collagen concentration alone does not fully account for the observed values. The HAPS/COL 80/20 formulation exhibited the highest density, 3.06 ± 0.01 g/cm^3^, despite containing more collagen than the 90/10 sample, 2.87 ± 0.005 g/cm^3^. Several factors may contribute to this behavior. First, variations in particle packing efficiency during measurement could lead to differences in apparent density. Second, collagen fibers may partially occupy microporous regions on the surface of HAPS particles, effectively reducing void volume and increasing the measured density beyond what would be expected from a simple additive mixture. This phenomenon has been observed in Hyder et al., 1992, where other composite systems where a lower-density phase fills pores of a higher-density matrix [6]. The higher standard deviation observed for the 80/20 formulation (0.01 g/cm^3^) compared to the other samples may also indicate slight heterogeneity in the mixture.

The non-linear behavior observed among the different formulations suggests that collagen content alone does not fully account for the measured values. In particular, the HAPS/COL 80/20 formulation exhibited the highest density among the three compositions, despite containing more collagen than the 90/10 sample. This indicates that additional structural factors may be influencing the final density. Variations in particle packing efficiency, the degree of interaction between mineral and organic phases, and possible differences in porosity generated during mixing could contribute to this behavior. Thus, the density reduction trend cannot be attributed solely to collagen concentration; instead, it likely results from a combination of intrinsic material densities and microstructural characteristics of each composite.

### 3.4. SEM-EDX

**Figure 9:**
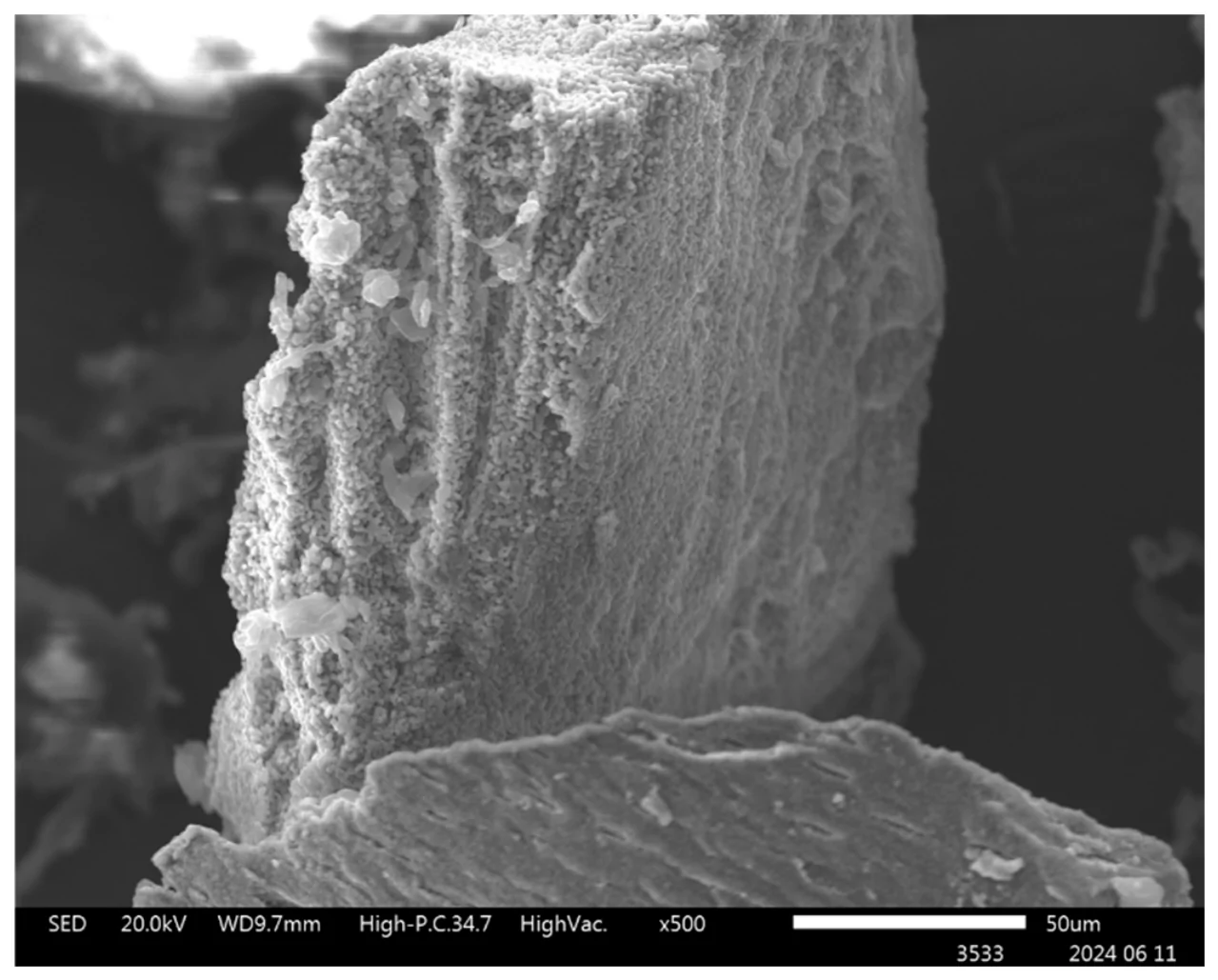
SEM of HAPS/COL 70/30.

**Figure 10:**
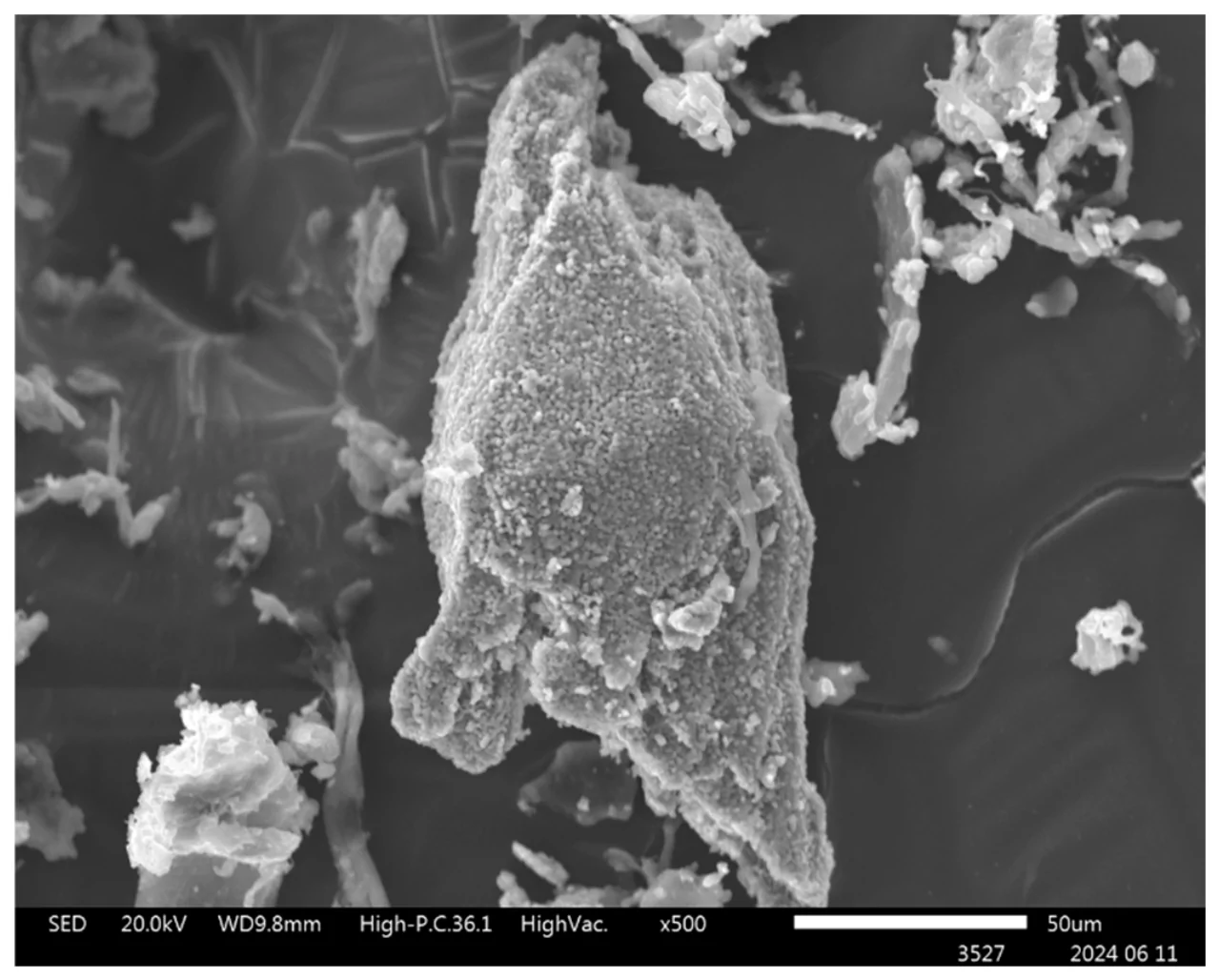
SEM of HAPS/COL 80/20.

**Figure 11:**
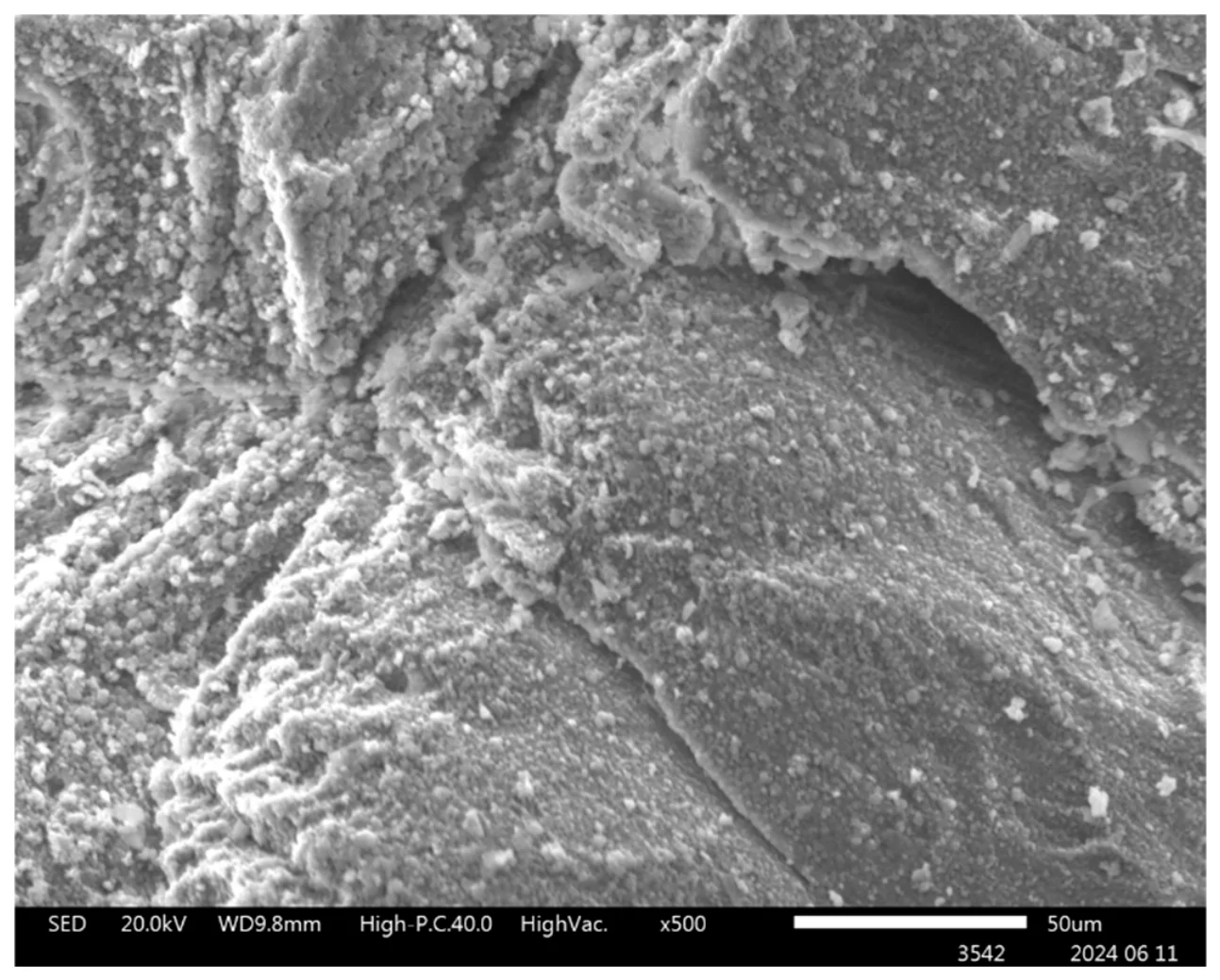
SEM of HAPS/COL 90/10.

**Figure 12:**
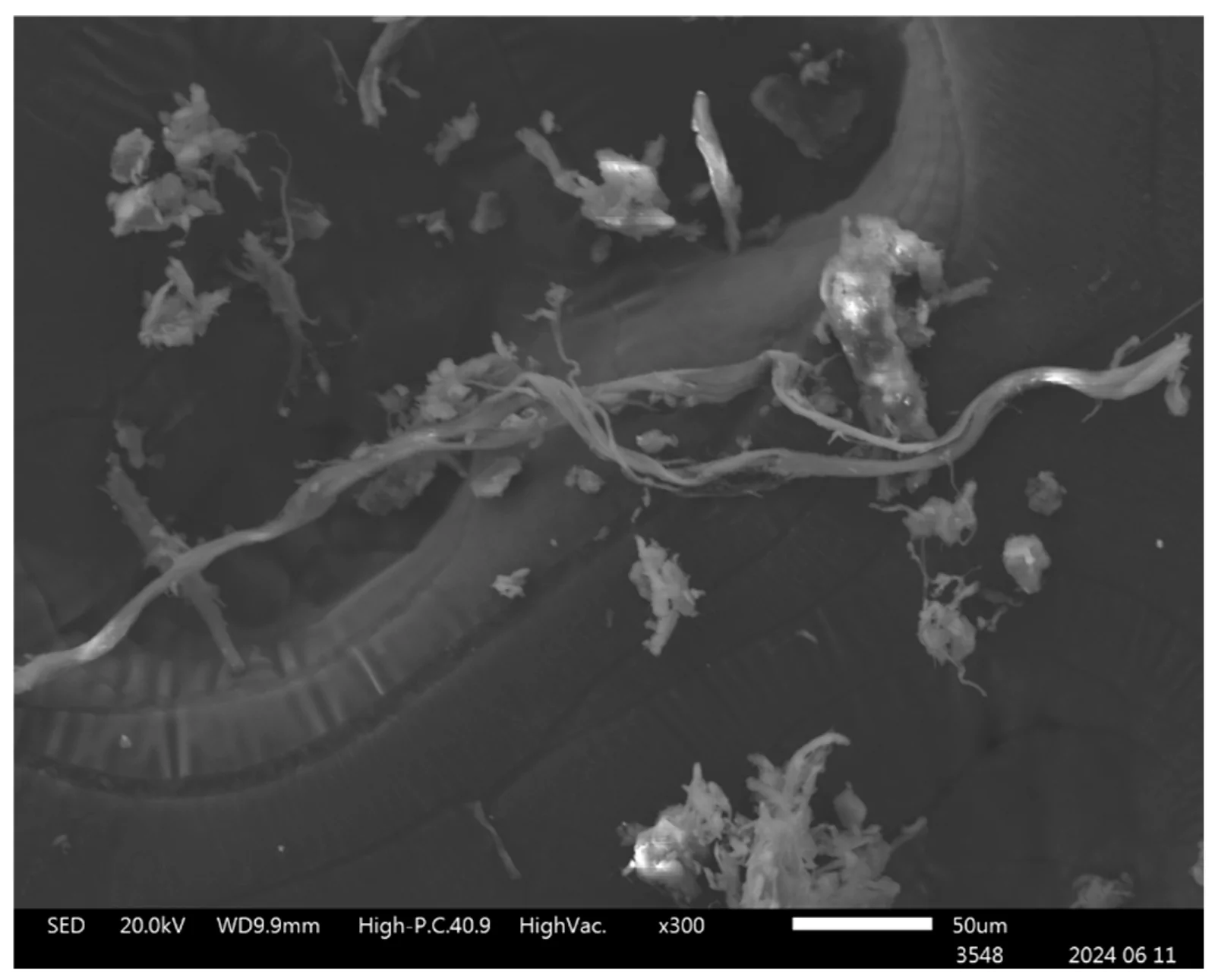
SEM of COL.

Figure 9, Figure 10, Figure 11 and Figure 12 show the SEM images obtained for each of the analyzed samples.

Additionally, the results of the EDX analysis are presented below.

In the SEM images corresponding to the three studied HAPS/COL ratios, the biomaterial exhibits a rough and porous surface as shown in Table 2. While HAPS and COL present markedly different physical characteristics on their own, their combination results in a blend where both phases contribute essential properties. HAPS, the inorganic component of the biomaterial, is rich in calcium and phosphorus, as confirmed by EDX analysis, yielding Ca/P ratios higher than 1.67, the theoretical value reported for this mineral [19]. In contrast, COL does not exhibit detectable levels of Ca and P, with concentrations below the equipment’s detection limit of 1000 ppm [19].

Nitrogen, a key element in organic matter, was consistently detected in atomic weight percentages ranging from 7 to 10% across all analyzed samples. This element is critical for the protein structure and serves as a relevant substrate for cell proliferation and lineage-specific differentiation.

Figure 12 shows the structure of type I collagen, appearing as elongated fibers. While the primary goal of this study is to develop a biomaterial that mimics both the composition and structure of human bone, the fibrillar morphology of COL also offers practical clinical advantages. Notably, collagen fibers easily agglomerate in the presence of leukocyte and platelet-rich fibrin (L-PRF), forming a cohesive unit known as “*sticky bone*”.

The observed morphology of the HAPS and COL is consistent with that reported by Rodrigues et al. [3], who described that composites of these materials generally exhibit a fibrillar appearance. In their study, collagen formed either locally oriented long fibers or irregular networks adhered to the surface of bone powder particles [3].

## 4. Discussion

The characterization of the HAPS/COL biomaterials revealed key structural and compositional insights that support their potential application in bone tissue engineering. XRD analysis demonstrated that the main crystalline phase in the mixtures was HAPS, with characteristic peaks corresponding to the (hkl) planes of this mineral. These peaks were consistent across all proportions studied (70/30, 80/20, and 90/10), confirming the stability of the HAPS phase regardless of COL content. In contrast, the XRD pattern for pure COL exhibited the typical features of an amorphous material, which aligns with the preservation of its native fibrillar structure. This lack of crystallinity is desirable, as it maintains the biological functionality of collagen, particularly its role in cellular adhesion and proliferation.

For TGA, COL showed significant mass loss between 200–300 °C, corresponding to a residual mass of 27.5% and maximum degradation temperature of 322.4 °C, in agreement with previous studies on collagen-containing bone structures [5]. In the mixture of materials, a gradual reduction in thermal stability was observed with increasing COL content, particularly in the HAPS/COL 70/30 sample. This is expected, as the organic phase degrades at lower temperatures compared to the mineral phase. Notably, HAPS alone exhibited no significant mass loss within this temperature range, reaffirming its high thermal resistance and inorganic nature. These findings highlight the importance of optimizing the organic/inorganic ratio to balance mechanical stability and biological performance.

The quantitative TGA data further reinforce this interpretation. The observed mass losses of 2.75%, 1.90%, and 1.60% for the 70/30, 80/20, and 90/10 formulations, respectively, correlate with their collagen content. The mass loss–collagen ratios for the 70/30 and 80/20 formulations were consistent (0.092–0.095), confirming that collagen is the primary source of thermal degradation within the 50–500 °C range analyzed, with the main mass loss event occurring between 200 and 400 °C. The HAPS phase exhibits no significant mass loss across the entire temperature range, reaffirming its thermal stability.

The slightly higher mass loss–collagen ratio for the 90/10 formulation, 0.160, compared to the other formulations, may reflect differences in the physical distribution of collagen within the mixtures. In formulations with lower collagen content, the organic phase is more thinly distributed over the HAPS particle surfaces, potentially exposing a greater proportion of collagen to thermal degradation. Alternatively, the higher ratio may result from minor sample heterogeneity, as the small sample size used in TGA (~9 mg) could amplify localized variations in composition. This interpretation is offered as a possible explanation for the observed behavior, however, direct evidence for the spatial distribution of collagen within the particulate mixtures was not obtained in the present study.

These findings suggest that while 10% collagen is sufficient to maintain the overall material structure, the thermal behavior of the organic phase may be influenced by its spatial distribution within the mixture. These values are consistent with those reported by Senra et al. [4] for hydroxyapatite–collagen composites, who observed similar mass loss trends corresponding to organic phase content. The absence of additional mass loss events suggests that no significant residual organic contaminants from the HAPS extraction process remain after calcination [4].

SEM imaging revealed that all HAPS/COL mixtures exhibited an irregular surface topography characterized by rough areas and the presence of pores of varying size. These observations are qualitative and suggest that the mixtures present surface features commonly associated with improved cellular attachment and mass transport; however, no quantitative analysis of porosity or pore distribution was performed in this study [20]. The micrographs also show regions where collagen and hydroxyapatite appear in proximity, although the images do not allow confirming true homogeneous integration or the absence of phase separation.

Additionally, while elongated collagen-like structures are visible in some samples, their distribution and continuity vary among images, even at the same magnification. Therefore, these features cannot be interpreted as uniform throughout the material. Given these limitations, the SEM results should be considered descriptive and representative of localized areas of the HAPS/COL mixtures rather than evidence of consistent morphological properties across the entire sample. The presence of elongated collagen fibers is a positive attribute that mimics the natural architecture of bone and facilitates cell guidance and scaffold cohesion, especially in clinical applications such as *sticky bone* formation.

EDX supported the compositional findings by detecting calcium and phosphorus in the mineral phase, with Ca/P ratios slightly above the theoretical value of 1.67. The consistent detection of nitrogen in all samples (7–10% by atomic weight) confirmed the presence of COL.

The non-linear density trend observed in the pycnometry data warrants further discussion. While the density values for all formulations were lower than that of pure HAPS, 3.16 g/cm^3^, and consistent with the incorporation of collagen, density ≈ 1.32 g/cm^3^, the absence of a monotonic decrease with increasing collagen content suggests that microstructural factors beyond simple mixing are at play. As proposed in the Results section, the partial filling of HAPS surface pores by collagen fibers could lead to a reduction in accessible void volume, thereby increasing the measured density in samples with moderate collagen content like in the case of the 80/20 sample. This phenomenon has been previously documented by Hyder et al. (1992), who observed that collagen can infiltrate porous mineral structures and alter density measurements [6]. Alternatively, variations in particle size distribution or agglomeration during sample preparation could influence packing efficiency. It should be emphasized that pycnometry measures skeletal density, excluding open pores, so any effect on porosity would directly influence the measured values.

Future studies employing quantitative micro-computed tomography (μ-CT) or focused ion beam scanning electron microscopy (FIB-SEM) would be valuable to confirm the microstructural basis for this behavior. μ-CT enables non-destructive 3D quantification of scaffold porosity, pore interconnectivity, and bone ingrowth, while FIB-SEM provides nanometer-scale resolution for visualizing mineral-organic interactions and interfacial structures [21,22].

Calcium ions on the HAPS surface may also act as bridges, chelating with carboxyl groups of collagen’s acidic residues; aspartic and glutamic acid [3]. These interactions could influence the cohesion of the particulate mixture, its resistance to washout in biological fluids, and its overall structural stability. Additionally, hydrogen bonding between the carbonyl groups of collagen’s peptide backbone and the hydroxyl groups of HAPS may contribute to phase adhesion [23]. However, the physicochemical techniques employed in the present study such as XRD, TGA and SEM-EDX are not designed to resolve the specific bonding interactions at the molecular level. Techniques such as Fourier-transform infrared spectroscopy (FTIR) or X-ray photoelectron spectroscopy (XPS) would be required to confirm the dominant interaction mechanisms and will be pursued in future work [24].

In previous studies, HAPS was obtained and characterized both physicochemically and biologically, demonstrating favorable properties for its application as a grafting material. In this work, we sought to add value to this bioproduct by incorporating collagen, considering that this component, as in natural bone tissue, plays a fundamental role in matrix structuring and promoting cell viability [2,14]. Based on the characterization performed—physicochemical analyses—it was observed that the presence of collagen does not significantly modify the intrinsic properties of HAPS. Even when varying the proportions of collagen in the formulations, the results remained consistent, indicating that the material maintains its expected behavior regardless of the added concentration. This finding is particularly relevant considering that collagen is currently a high-value commodity. Therefore, using the lowest proportion evaluated represents an efficient and cost-effective alternative, as it allows for obtaining HAPS/COL mixtures with the desired characteristics without compromising graft performance. Taken together, these results suggest that the moderate addition of collagen is sufficient to enhance the value of the material, while maintaining its economic viability for future applications.

One of the weaknesses of this research lies in the choice of collagen cross-linking method. Classical cross-linking methods are based on physical, chemical, and enzymatic approaches [25]. In this case, the choice of ultraviolet light was made with the idea that the final product (HAPS/COL) would be used in biomedical applications. Therefore, alternatives involving the addition of chemical agents to the structure or that could cause collagen denaturation were ruled out. However, further study of the most suitable methodology is needed to successfully complete this stage, as this project lacked specialized analyses that would allow for a precise evaluation of the collagen cross-linking present.

Future studies will be necessary to evaluate the in vitro and in vivo performance of these mixtures, with emphasis on their effects on osteoblast and osteoclast activity, as well as their long-term resorption behavior and remodeling capacity.

## 5. Conclusions

The HAPS/COL particulate mixtures prepared at ratios of 70/30, 80/20, and 90/10 were successfully characterized through physicochemical analyses, which confirmed the preservation of the hydroxyapatite phase and the influence of collagen incorporation on selected material properties. XRD, TGA, pycnometry, and SEM-EDX results indicated that all formulations maintained the crystalline structure of HAPS, while the addition of collagen contributed to a reduction in measured density. The Ca/P ratios obtained were consistently higher than that of stoichiometric hydroxyapatite, a feature commonly reported for biologically derived mineral sources.

The selection of the three HAPS/COL proportions (70/30, 80/20, and 90/10) was intended to determine whether varying the relative amounts of mineral and organic components would produce marked differences in the physicochemical outcomes. Although the characterization results showed some variations among formulations, the overall influence of collagen content on the measured parameters was not substantial. From a compositional standpoint, the 70/30 mixture is expected to be the most representative of human bone, as it approximates the natural balance between mineral and organic phases; however, this consideration is theoretical and cannot be confirmed solely from the present data. While the physicochemical analyses provided a useful foundation for understanding the behavior of these mixtures, biological assays will be essential to validate their biocompatibility and confirm their suitability for future biomedical applications.

## Figures and Tables

**Table 1:** Density of the biomaterials analyzed.

Sample	Density (g/cm^3^)	Specific Volume (cm^3^/g)
HAPS/COL 70/30	2.66 ± 0.03	0.35 ± 0.003
HAPS/COL 80/20	3.06 ± 0.01	0.31 ± 0.004
HAPS/COL 90/10	2.87 ± 0.005	0.33 ± 0.001

**Table 2:** EDX of the analyzed biomaterials.

Chemical Symbol/Atomic Weight (At%)	HAPS/COL 70/30	HAPS/COL 80/20	HAPS/COL 90/10
N	8.5 ± 0.56	8.3 ± 2.1	9.5 ± 1.01
Ca	5.93 ± 0.96	6.76 ± 4.54	7.53 ± 0.55
P	2.86 ± 0.49	3.23 ± 1.36	3.86 ± 0.61
Ca/P	2.07 ± 0.15	1.98 ± 0.53	1.99 ± 0.46

## Data Availability

The original contributions presented in this study are included in the article. Further inquiries can be directed to the corresponding author.

## Abbreviations

The following abbreviations are used in this manuscript:

HAPS	hydroxyapatite derived from salmon bone
COL	bovine type I collagen
XRD	X-ray diffraction (XRD)
TGA	thermogravimetric analysis
SEM-EDX	scanning electron microscopy with energy-dispersive X-ray spectroscopy
